# Patient Experience and Satisfaction with Orthopedic Services at a Community (Rural) Setting Hospital—How Is It Different from Urban Setting

**DOI:** 10.3390/jmahp12030017

**Published:** 2024-08-05

**Authors:** Khalid Hasan, Shahin Kayum

**Affiliations:** 1 Department of Orthopaedic Surgery, Virginia Commonwealth University, Richmond, VA 23284, USA; 2 Department of Orthopedics, University of Toronto, Toronto, ON M5T 1P5, Canada

**Keywords:** patient satisfaction, patient experience, access to healthcare, community setting, rural, urban

## Abstract

Patient experience and satisfaction are the keystones in evaluating the effectiveness of clinical care in musculoskeletal medicine. Although all orthopedic settings work on the same principles of providing safe and quality health care, community hospitals represent a unique environment. There may be key differences with regard to patient experience between these settings. Accessibility to care, choices of provider, personalized care, availability of and access to resources, cultural and social variances, and waiting times are a few of the many elements that may impact patient experience and satisfaction. This narrative review aims to explore the core differences in these settings and how they can reflect on patient experience and satisfaction.

## 1. Introduction

Patient experience and satisfaction are critical in determining the efficacy of musculoskeletal care. They can be considerably influenced by the type of healthcare setting. Providing orthopedic care at rural or community-based hospitals can be significantly more challenging compared to urban settings due to multiple factors. Orthopedics is still technically considered one of the forms of specialized care and is not part of the primary care [1]. Moreover, most community hospitals provide general orthopedic care and lack subspecialized care [2,3]. This results in a limited scope of care for the patients visiting these hospitals compared to urban settings and directly impacts their experience and satisfaction.

The United States Bureau of the Census defines urban and rural areas based on housing and population. According to the Bureau, rural areas comprise open country and settlements with fewer than 2000 housing units and 5000 residents, whereas the urban areas involve areas with 2000 or more housing units or 5000 or more residents [4].

There are multiple factors that impact health care provision for patients in rural versus urban settings. This ultimately reflects on patient experience and satisfaction with the care provided. While patient satisfaction and experience are well-studied topics, it is noticed that they are more generalized, and these differences are not considered. The authors believe that when patient experience and satisfaction are gauged, they should be reflective of the type of healthcare setting in which they have received the orthopedic care. The article narrates and highlights these differences in the United States of America and provides a possible solution to address them.

## 2. Differences in Rural versus Urban Setting Healthcare Settings 

Patient experience with orthopedic services can be influenced by a multitude of factors, whether in a rural or urban setting. Some of the key differences that influence patient experience within the United States are described here (Table 1).

### 2.1. Access to Care

The ease with which patients can access orthopedic services, including the availability of appointments, waiting times, and the proximity of the facility to the patient’s home are some of the key components in this regard. It has been demonstrated that only 30% of rural hospitals have orthopedic surgeons available [5]. Moreover, rural patients who reside on farms, ranches, reservations, and frontiers often have to travel long distances to reach a healthcare provider and sometimes may need to travel hundreds of miles to get to the desired professional [3,6]. This often results not only in a delay of the initial presentation but also impacts follow-up visits, which are less likely to be attended in a timely fashion. Several techniques, including e-consults, telemedicine, and virtual clinics, have been tried in the past with variable results. This is in part due to the lack of adequate internet access in rural communities compared to the urban areas [7]. Thirty-nine percent of rural Americans lack adequate internet access, versus four percent of the urban population, as reported in a report by the Federal Communication Commission [8].

### 2.2. Quality of Care

The level of skill and expertise of orthopedic services can differ significantly between rural and urban settings. It is estimated that only 9% of orthopedic surgeons practice in the rural settings in the USA, serving approximately 20% of the country’s population [9,10]. In a study, it has been demonstrated that there is a 32% greater chance of death following a hip fracture in a rural setting compared to an urban setting [11].

### 2.3. Limited Resources

Rural hospitals are usually of smaller capacity and less likely to offer surgical treatment compared to urban hospitals [12]. Limited resources may include lack of human resources, equipment, or supplies needed for an optimal outcome. Some of the key components of these resources include anesthesiologists, therapists, the latest surgical equipment, wound care, and other ancillary support. If the patient receives less than optimal orthopedic care due to shortage or lack of resources, it is likely to negatively impact patient experience and satisfaction with the provided care and the hospital. For instance, a patient with a wrist fracture received a reduction with less-than-optimal pain control due to the unavailability of a nighttime anesthetist. The intraarticular fracture required surgical fixation, and the surgeon had to use an implant which was not designed for this fracture configuration as the fracture-specific implant was not obtainable. This was followed by hand and wrist stiffness as a hand therapist was not available to initiate early therapy.

### 2.4. Patient Population

It has been demonstrated that the rural population has a higher incidence of chronic illnesses, including uncontrolled blood pressure, chronic obstructive pulmonary disease, cancer, diabetes mellitus, and obesity. Moreover, the rural population tends to have a higher rate of unhealthy habits compared to the urban population, such as smoking, alcohol and drug abuse, and lack of exercise [13,14]. A large proportion of the aging population live in the rural community. This makes the rural population more vulnerable to not receiving proper orthopedic care in a timely fashion. The ratio of healthcare providers in rural areas per 10,000 population is significantly lower in rural areas compared to urban areas [15,16].

### 2.5. Personalized Care

In a community setting hospital, it is anticipated that the patients may receive personalized care and build a stronger physician–patient relationship due to a smaller number of patients. In addition, it is also likely that the patients are more socially connected with the healthcare provider for the same reason. Similarly, it is reported that physicians feel more deeply connected with their patients in rural communities [17].

### 2.6. Delay in Presentation

In rural settings, the need for specialized orthopedic care can occasionally be underestimated by initial non-orthopedic provider assessment since it is very much dependent on the primary provider’s training, knowledge and access to peer-to-peer consultation with an orthopedic surgeon. Additionally, rural areas have lower chances of having an organized transportation infrastructure and resources, which can cause a delay in healthcare access [7].

### 2.7. Post-Treatment Support

Post-treatment care can be much more challenging in rural settings. This care includes outpatient therapy and rehabilitation centers, which are much scarcer in rural settings compared to urban areas. This can impact optimal outcomes in patients.

### 2.8. Patient Choice of Provider and Need for Subspeciality

Due to limited resources and orthopedic providers in the community settings, most patients do not have the choice to choose between providers. Similarly, lack of subspeciality often leaves these patients in the care of the available orthopedic provider. This can be critical, especially with the vulnerable patient population, such as pediatric patients requiring pediatric orthopedic services, elderly patients in need of geriatric orthopedic specialists, and even pregnant woman who may require orthopedic services but also require advanced mother and neonatal/childcare services to be available within the same settings.

**Table 1:** Summary of differences between rural and urban settings.

	RURAL	URBAN
**Access to care**	Limited access but shorter waiting times	Better access but possibly longer waiting times
**Quality of care**	Often inadequate	Superior quality of care compared to rural setting
**Limited resources**	Likely to suffer from limited resources	Less likely to be problem compared to rural hospitals
**Patient population**	Higher number of aging and unhealthy population	More diverse population
**Personalized care**	Better seen in rural setting	Less prevalent in urban hospitals
**Delay in presentation**	More commonly encountered	Possible but chances are less likely
**Post treatment support**	Often deficient	More availability and numerous possibilities
**Patient choice of provider and need for subspeciality**	Often limited	Less likely to be a problem

## 3. Discussion

Patient experience and satisfaction are crucial and often used metrics to assess the caliber of medical service. This is true for any specialty, including orthopedics. Clinical results, patient retention, and medical malpractice lawsuits are all impacted by patient satisfaction. It has an impact on the prompt, effective, and patient-centered provision of high-quality medical treatment [18]. Patient satisfaction has also been correlated with the quality of medical care or hospital performance [19].

Patient experience and satisfaction with orthopedic services are influenced by numerous factors. These factors can differ greatly in a community setting hospital compared to urban hospitals. It is imperative to identify the similarities and dissimilarities between these two systems to provide better care which is tailored to individual patients. Tailored healthcare, a concept which has existed since 4000 BC, has repeatedly been shown to improve patient outcomes and elevate the overall experience [20].

As highlighted, differences and disparities exist between these two healthcare settings. These variances directly and indirectly influence patient satisfaction and experience. For instance, patients in the community setting may not receive critical orthopedic care in a timely manner, resulting in a poorer outcome. This can happen even if adequate orthopedic service is provided in a timely fashion. An example of this can be a Maisonneuve fracture that was treated as a sprain during initial primary provider assessment. Due to lack of subspecialized training at the initial provider end, difficulty in transportation, and the perceived nonurgency of the situation, the patient is not seen in the orthopedic office until a few weeks have passed. This can result in a poorer overall outcome even if the patient is treated surgically in an appropriate manner after presenting in the orthopedic office. In a different scenario, a comminuted elbow fracture dislocation gets operated on by an orthopedic surgeon who does not usually perform these types of complex surgeries but was obligated to do so due to a lack of subspecialized care in the vicinity. In addition, there was no availability of a physical or occupational therapist within a reasonable distance of the patient’s residence for post-operative care and rehabilitation. These is highly likely to result in less-than-optimal outcomes, thus negatively impacting patient experience and satisfaction.

It is also important to acknowledge that these factors influencing patient experience often coexist and frequently result in a collective impact. Patient experience is often defined as a range of collaborations that patients undergo during their healthcare visit [21]. This is also true for the orthopedic services provided. In this array of multiple constituents, the chances of a less-than-desirable experience becomes higher in a rural setting. It is therefore imperative that when gauging patient experience, these factors are considered.

The literature is very variable when it comes to patient experience in rural and urban settings. For instance, the readmission rate after total hip arthroplasty is classically thought to be higher in rural settings, but a recent study has noticed that urban patients are more likely to visit the emergency department within 30 days and to be readmitted within 90 days after undergoing a total hip replacement [22]. Similarly, patient-reported outcome measures (PROMs) are also not very well defined in the existing literature. Some studies have shown better PROMs statistically in the rural safety-net hospitals but failed to reach minimal clinically important difference [22,23]. Another study, however, found higher dissatisfaction in the rural patients after undergoing joint replacement surgery [24].

It is also debated that patient experience and satisfaction, although interchangeable, are two separate entities. Patient satisfaction is usually defined as patient actual care compared to perceived care or expectation [21]. It can be argued that patient satisfaction may be better in a rural setting despite less-than-optimal experience due to a lower level of expectation. In a study comparing patient perception of healthcare in rural and urban hospitals, it was found that rural hospitals performed better and that the patients had experienced more improvements in several outcome measures between 2014 and 2019, but the rural hospitals were still less likely to be recommended by the patients compared to their urban counterparts [25].

The next most important question is how patients’ experience and satisfaction can be improved in a rural setting or in community hospitals. This needs to be addressed at multiple levels simultaneously.

The hospital administration can work at their end, and steps can be taken that include the recruitment of more specialist orthopedic surgeons, the procurement of an advanced and up-to-date surgical inventory, and the addition of ancillary services like therapy and rehabilitation to the practice [7]. Unfortunately, most of these require significant resources and financial support. Many rural hospitals are unable to afford these. A reasonable option in these situations is to affiliate with a higher-level care center or the nearest urban hospital. In this way, the rural hospital can collaborate with the urban hospital and will even allow patients to be transferred to subspecialized orthopedic care if required.

The rural hospital can consider the purchase of a hospital-owned bus/van. Although it broadly falls into procurement of new material, it has much more widespread benefits. Hospital-owned transport can not only help in reaching out to a community patient in a timely fashion, it can also be utilized to transport a patient to a different facility if deemed necessary. This will also help in improved patient access to the hospital services as rural communities do prefer to obtain healthcare locally if possible [26,27].

Another important aspect is the educating of patients and providers. This includes educational seminars as well as community outreach meetings by lead orthopedic surgeons to enlighten non-orthopedic providers and prospective patients regarding the most common orthopedically related problems and injuries, and how to address them effectively and promptly. This also includes the updated training of existing orthopedic surgeons.

One more aspect to highlight here is the out-of-pocket expense for orthopedic healthcare provision. The rural population is expected to spend slightly more compared to the urban residents. This can be attributed to the higher chance of being uninsured as well as the higher incidence of chronic diseases and risky health care behavior [28]. This can be addressed with educational seminars and community orientation sessions.

Orthopedic providers who serve at rural or community care hospitals also need to understand their limitations. They should be open with patients about what they can and cannot do. They should be willing to collaborate and to refer patients to appropriate subspecialists if clinically indicated. This not only includes patients that may require ancillary non-orthopedic care unavailable at that location but also incorporates surgeon experience and level of comfort with certain procedures. The patient deserves to be treated in the best available location and in the best available surgical hands even if that comes through an outside hospital and/or provider. This can sometimes be fulfilled by inviting an orthopedic surgeon who is qualified to serve the specific needs of the patient in the community [29]. Another possibility is video conferencing with the specialist orthopedic provider, which can boost patient confidence and overall satisfaction [30].

There are certain other aspects to consider in community settings. Smaller rural communities are often well connected compared to larger urban neighborhoods. Word of mouth plays a much more significant role in these communities. Events like townhall meetings and church/religious gathering are much widely attended and may include discussion about orthopedic services provided at the community hospital by a particular provider. Other strategies, which include telehealth, e-consult, outreach clinics, and virtual clinics, can be introduced to address these disparities regarding getting proper orthopedic care [7].

The literature has demonstrated that a team-based approach has a strong and positive impact on patient experience [31]. This can sometimes be more complicated in the rural hospitals due to smaller settings and limited resources. Moreover, many times, primary care can be based at a remote location in the rural setting, whereas the orthopedic services are centered at the main hospital. It is important that the communication between these offices is conducted in real time, and the patient should experience well-coordinated and team-based care, which will lead to better outcomes.

It is imperative that patient experience and satisfaction are addressed and more specifically tailored to the local population’s needs and preferences and are not generalized. A lot of the research that investigated patient satisfaction and experience did not differentiate between these two settings. Unfortunately, about 2/3rd of rural communities still do not have access to a local orthopedic surgeon, which makes the rural community orthopedics and the patient experience distinct, and this should be dealt with [32,33].

Although this review focuses on the health systems in the United States, it is necessary to mention that these differences occur in other health systems throughout the world. As different countries work on different principles of healthcare provision, these differences can sometimes be substantial. In a study measuring patient satisfaction and experience in the United States, the United Kingdom, Germany, and Scandinavian countries, it was noted that most countries have a well-established system of gathering patient outcome data, but the comparability of these data is challenging due to lack of unanimity within the questionnaires [34]. It is also important to acknowledge that these differences can vary among countries at different stages of development. Most of these data come from developed nations where the health system is also well developed. In low-income countries and developing nations with less robust healthcare systems, the differences between the rural and urban settings can be considerably significant. In a developing nation, the healthcare resources distribution disparity ratio can be up to 17 times between the urban and rural healthcare settings [35]. As patient satisfaction and experience are directly proportional with healthcare resources, higher difference in these resources will ultimately mean lower patient satisfaction scores with orthopedic services in the rural settings.

## 4. Conclusions

It is important to tailor and assess patient experience and satisfaction based on the hospital setting. Differences exist between urban and rural patient expectations, which can directly impact the outcome. Steps can be taken to improve the existing setups. However, large multicenter prospective studies are required to study and explore this aspect of patient experience and satisfaction.

## Data Availability

No new data were created or analyzed in this study. Data sharing is not applicable to this article.
